# Quadratic Forms in Random Matrices with Applications in Spectrum Sensing

**DOI:** 10.3390/e27010063

**Published:** 2025-01-12

**Authors:** Daniel Gaetano Riviello, Giusi Alfano, Roberto Garello

**Affiliations:** 1 CNR-IEIIT, Istituto di Elettronica e di Ingegneria dell’Informazione e delle Telecomunicazioni, Consiglio Nazionale delle Ricerche, 10129 Turin, Italy; daniel.riviello@cnr.it; 2 Department of Electronics and Telecommunications (DET), Politecnico di Torino, 10129 Turin, Italy; d020860@polito.it

**Keywords:** spectrum sensing, quadratic forms, multi-antenna, random matrix theory, cognitive radios, 6G

## Abstract

Quadratic forms with random kernel matrices are ubiquitous in applications of multivariate statistics, ranging from signal processing to time series analysis, biomedical systems design, wireless communications performance analysis, and other fields. Their statistical characterization is crucial to both design guideline formulation and efficient computation of performance indices. To this end, random matrix theory can be successfully exploited. In particular, recent advancements in spectral characterization of finite-dimensional random matrices from the so-called *polynomial ensembles* allow for the analysis of several scenarios of interest in wireless communications and signal processing. In this work, we focus on the characterization of quadratic forms in unit-norm vectors, with unitarily invariant random kernel matrices, and we also provide some approximate but numerically accurate results concerning a non-unitarily invariant kernel matrix. Simulations are run with reference to a peculiar application scenario, the so-called spectrum sensing for wireless communications. Closed-form expressions for the moment generating function of the quadratic forms of interest are provided; this will pave the way to an analytical performance analysis of some spectrum sensing schemes, and will potentially assist in the rate analysis of some multi-antenna systems.

## 1. Introduction

The statistical characterization of Quadratic Forms (QFs) with random kernel matrices is an ubiquitous task in applied multivariate statistics (see, e.g., [1]). In the realm of wireless communications, such QFs appear in a wide range of topics, such as the sum-rate analysis and spectral efficiency computation of multi-user systems (see ([2] and references therein)). An ergodic capacity analysis of single-user multi-antenna wireless links relies on the knowledge of the probability density function (pdf) of QFs in Gaussian random matrices [3]; outage probability in multiuser beamforming can be computed, exploiting indefinite QFs [4]. On top of that, recasting a Rayleigh quotient in terms of an indefinite QF can lead to the quantification of the spectral efficiency of linear receivers ([5,6] and references therein).

Often, test statistics for sensing applications reduce to or involve QFs in random matrices (see, for example, the case of energy detection techniques [7]). Due to the vastness of the field, our literature review is far from being exhaustive. We only remark that, for our purposes, we have mostly focused on results assuming the involved matrices to be of finite size. Otherwise, in the traditional approach of random matrix theory, where the number of rows and columns of the matrices at hand are assumed to diverge at the same rate, the study of QFs with random kernel matrices can be seen as a particular case of the study of linear spectral statistics; the interested reader is referred to ([8] and references therein) and successive developments.

Our work focuses on the characterization of scalar QFs in unit-norm vectors, with Hermitian random kernel matrices being unitarily invariant in the sense of ([2] Def. 2.6). This, in turn, implies that the joint density of the non-zero eigenvalues of such matrices is independent from the density of the corresponding eigenvectors. While such an assumption might seem overly restrictive, it still encompasses random Hermitian matrix models widely adopted in radio channel modeling, statistical testing, Spectrum Sensing (SS), such as, e.g., complex central uncorrelated Wishart matrices (see ([2] and references therein), [9]), complex, uncorrelated non-central Wishart matrices [10] whose non-centrality parameter is a multiple of the identity matrix, and the full-rank Gram matrix of the product of a finite number of complex zero-mean Gaussian random matrices with independent and identically distributed entries [11]. While uncorrelated central Wishart matrices provide a canonical model for single-user multi-antenna links affected by Rayleigh fading [12], the unitarily invariant non-central Wishart is commonly exploited in likelihood ratio tests [13]. In turn, products of independent Gaussian matrices model both multiple scattering phenomena (see, again, [11], and also [14,15]), and, when the random matrix factors are interleaved with suitably chosen diagonal matrices, multi-hop relay communications ([16] and references therein).

The paper is structured as follows: Section 2 contains the main result, i.e., the statistical characterization of a scalar QF in a unitarily invariant random kernel matrix, with a unit-norm vector. Statistics thereof are given in terms of the Moment Generating Function (MGF). Particularization for two cases of practical interest, namely the uncorrelated central Wishart and the product of independent Gaussian matrices, is then reported in Section 2.2, along with the analytical expression of the pdf of the related QF, whenever the MGF is amenable to analytically handy inversion. A numerically accurate expression for the MGF of a QF in a non-unitarily invariant random matrix, obtained with a suitable approximation, is also presented. Within the same section, numerical results are presented, displaying the statistical behavior of our expressions with reference to the set of kernel matrices at hand, for varying matrix sizes and/or numbers of involved matrix factors. Section 3 is devoted to Cognitive Radio Network (CRN) applications, where the performance of a secondary-data-aided SS scheme is discussed, and its test statistics is approximated by a QF in unitarily invariant matrices, exploiting our newly derived expression. Then, conclusions are drawn in Section 4, along with discussions on future developments and ongoing work.

## 2. Quadratic Form Statistics

This section contains our main result; after a detailed description of the mathematical framework, a proposition, along with its proof, and therefore some corollaries, referring to analytical models of relevance in multivariate statistics and signal processing, are presented.

### 2.1. Mathematical Framework

Given a unit-norm vector v of length *K*, with complex entries, and a Hermitian random matrix A of size *K*, we focus on the random variable $W=vAv^{H}$. The statistical characterization of *W* depends on the spectral statistics of $A=U\Lambda U^{H}$, where Λ denotes the diagonal matrix of the eigenvalues of A, for which, without loss of generality, the order $\lambda_{1}>\ldots >\lambda_{K}\geq 0$ is assumed, and U is the matrix of the corresponding eigenvectors. Notice that both U and Λ are themselves random matrices, with probability laws depending on the statistics of the entries of A. Hereafter, we focus on *unitarily invariant* random matrices (see ([2] Def. 2.6)); this assumption implies that U and Λ are statistically independent random matrices, and that U follows the Haar distribution ([2] Lemma 2.6), i.e., it is uniformly distributed on U(K), the unitary group of size *K*. As to the eigenvalue statistics, we focus on so-called *polynomial ensembles* (PE), namely sets of matrices whose eigenvalues are jointly distributed according to a determinantal law, where

(1) $$f\left(\Lambda \right)=c|\varphi_{i}\left(\lambda_{j}\right)|\prod_{1\leq i<j\leq K}\left(\lambda_{i}-\lambda_{j}\right),$$

where *c* is a suitable normalizing constant, $\varphi_{i}\left(\cdot \right)$, i=1,…,K are a set of (not necessarily different) scalar functions, each evaluated at a single eigenvalue, and $\prod_{1\leq i<j\leq K}\left(\lambda_{i}-\lambda_{j}\right)$ is the Vandermonde determinant [[17], (0.9.11.2)] of Λ.

With these assumptions in mind, we are able to state our main result in the following proposition:

**Proposition** **1.** *Given a Hermitian, unitarily invariant matrix A, of size K, from a polynomial ensemble, and a unit-norm vector of length K, say v, the MGF of the QF $W=v^{H}Av$ can be written as*

(2) $$\begin{array}{c} \Phi_{W}\left(s\right)=C\frac{\left|\Upsilon (s)\right|}{s^{K-1}}, \end{array}$$

*with C as a normalizing constant, and the K×K matrix Υ(s) having entries*

$${\left[\Upsilon \left(s\right)\right]}_{i,j}=\int_{0}^{+\infty }\lambda^{K-j}\varphi_{i}\left(\lambda \right)d\lambda .$$

**Proof.**  In terms of the spectral decomposition of A, the random variable of interest can be rewritten as

$$W=v^{H}U\Lambda U^{H}v.$$

Therefore, the MGF thereof can be expressed as

(3) $$\begin{array}{c} \Phi_{W}\left(s\right)=E\left[e^{-sW}\right]=E\left[\exp\left(-\mathrm{tr}\left(svv^{H}U\Lambda U^{H}\right)\right)\right], \end{array}$$

where the expectation is to be taken jointly with regard to both U and Λ. Due to the postulated statistical independence between U and Λ, and to the uniform distribution of the eigenvector matrix on its group, we can compute first the average of (3) with regard to U, obtaining, by virtue of the Harish–Chandra–Itzykon–Zuber (HCIZ) integral ([10] Equation (92)), the conditional MGF, given Λ, namely

(4) ΦW|Λ(s)=F00−svvH,Λ=(K−1)!Θ(s)(−s)K−1∏i<j(λi−λj),

where the K×K matrix Θ(s) has the entries

$${\left[\Theta \left(s\right)\right]}_{i,j}=\left\{\begin{array}{c} \exp\left(-s\lambda_{i}\right),\;i=1,\ldots ,K,\;j=1,\\ \lambda_{i}^{K-j}\;i=1,\ldots ,K,\;j=2,\ldots ,K, \end{array}\right.$$

and the hypergeometric function F00·,· of two Hermitian matrix arguments with different ranks (see, e.g., ([18] Appendix B) for a detailed definition) has been expressed as a ratio of determinants, exploiting ([19] Corollary I).

Subsequently, to remove the dependence on Λ, distributed according to (1), the joint *unordered* eigenvalue distribution is first expressed, i.e., the law given by

(5) $$f\left(\Lambda \right)=\frac{c}{K!}\left|\varphi_{i}\left(\lambda_{j}\right)\right|\prod_{1\leq i<j\leq K}\left(\lambda_{i}-\lambda_{j}\right),$$

on ${\left[0,+\infty \right)}^{K}$ is considered. Then, the following integral is computed, with the help of ([9] Corollary I)

(6) $$\begin{array}{c} \Phi_{W}\left(s\right)=\frac{c(K-1)!}{{\left(-s\right)}^{K-1}K!}\int_{{\left[0,+\infty \right)}^{K}}\left|\Theta (s)\right|\left|\varphi_{i}\left(\lambda_{j}\right)\right|d\Lambda , \end{array}$$

and the statement of the proposition, in terms of (2), follows. □

### 2.2. Statistics for Given Kernel Matrices

Proposition 1 can be particularized, upon the selection of a specific matrix whose statistics satisfy the assumptions. In the remainder of this section, we provide analytical expressions of the QF statistics for two random matrix models widely adopted in wireless communications and signal processing settings: the complex central uncorrelated Wishart, and the Hermitian product of independent complex zero-mean Gaussian matrices, in the following two corollaries.

**Corollary** **1.** *Assuming the kernel matrix to be complex central uncorrelated Wishart-distributed, with N≥K degrees of freedom, i.e.,*

(7) $$\begin{array}{c} p\left(A\right)=\frac{{\left|A\right|}^{N-K}e^{-\mathrm{tr}(A)}}{\prod_{\ell =1}^{K}{\left(\sqrt{\pi }\right)}^{K-1}\left(N-\ell \right)!}, \end{array}$$

*the MGF of the QF is obtained from* (2)*, with*

$${\left[\Upsilon \left(s\right)\right]}_{i,j}=\left\{\begin{array}{c} \frac{(N-j)!}{{\left(1+s\right)}^{N-j+1}},\;i=1,\;j=1,\ldots ,K\\ (N+K-i-j)!\;\;i=2,\ldots ,K,\;j=1,\ldots ,K \end{array}\right.$$

*while*

(8) $$C=\frac{(K-1)!}{\prod_{\ell =1}^{K}\left(K-\ell \right)!\left(N-\ell \right)!}.$$

**Proof.**  To compute the expectation in (3), (7) is transformed, exploiting the Jacobian of eigenvalue–eigenvector decomposition for $A=U\Lambda U^{H}$ (see ([20] Equation (6)) and ([9] Equation (6))). This leads to

(9) $$\begin{array}{c} p\left(A\right)dA=\frac{\prod_{\ell =1}^{K}\lambda_{\ell }^{N-K}e^{-\lambda_{\ell }}\prod_{i<j}{\left(\lambda_{i}-\lambda_{j}\right)}^{2}}{\prod_{\ell =1}^{K}\left(K-\ell \right)!\left(N-\ell \right)!}d\Lambda dU, \end{array}$$

apparently uniform with regard to U. As to the ordered eigenvalues, their joint law on the subset of $R^{K}$ defined by $\left\{\lambda_{1}\geq \ldots \geq \lambda_{K}\geq 0\right\}$ can be rewritten as

(10) $$\begin{array}{c} p\left(\Lambda \right)d\Lambda =\frac{|\varphi_{i}\left(\lambda_{j}\right)|\prod_{i<j}\left(\lambda_{i}-\lambda_{j}\right)}{\prod_{\ell =1}^{K}\left(K-\ell \right)!\left(N-\ell \right)!}d\Lambda , \end{array}$$

with $\varphi_{i}\left(\lambda_{j}\right)=\lambda_{j}^{N-i}e^{-\lambda_{j}}$. Therefore, proposition 1 can be applied, with

$${\left[\Upsilon \left(s\right)\right]}_{i,j}=\left\{\begin{array}{c} \int_{0}^{+\infty }\lambda^{N-i}\exp\left(-(s+1)\lambda \right)d\lambda ,\;i=1,\ldots ,K,\;j=1,\\ \int_{0}^{+\infty }\lambda_{i}^{N+K-i-j}e^{-\lambda }d\lambda \;i=1,\ldots ,K,\;j=2,\ldots ,K. \end{array}\right.$$

Closed-form expressions of the entries of Υ(s), as per the statement of our Corollary, are recovered resorting to ([21] 3.381.4).

□

Upon Laplace inversion of the MGF, the PDF of *W* is retrieved, namely

(11) fW(t)=C∑j=1Kκj(N−j)!tN+K−j−1(N+K−j−1)!e−tF11K−1;N+K−j;t,t>0,

with F11a;b;z being the confluent hypergeometric function ([22] Ch.13), $\kappa_{j}$ the co-factor of the (1,j)-th entry of Υ(s), and *C* as per (8).

**Figure 1:**
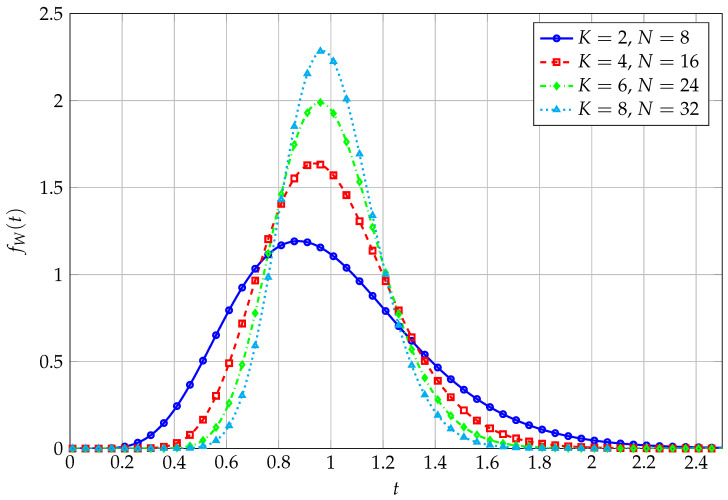
PDF of QF with uncorrelated Wishart kernel matrix for varying *K* and *N*.

Analytical (11) versus simulated PDFs of *W*, for varying values of *K* and *N*, are reported in Figure 1. As both *K* and *N* increase, with a fixed ratio (e.g., K/N=0.25 in this Figure), PDFs become more and more peaked, as expected.

It would be very useful in several applications to be able to extend the analytical results holding for uncorrelated Wishart matrices to the correlated case. Unfortunately, even in the simplest setting (full-rank Wishart matrix with single-sided Kronecker correlation [2]), a correlated Wishart matrix is no longer unitarily invariant. However, although our proposition cannot be rigorously applied to the general case of complex correlated Wishart matrices, a very accurate approximation of the MGF of the QF when the kernel matrix is a semi-correlated Wishart, in the sense of ([23] Thm I), can still be obtained. To compute such an approximated expression, we capitalize on the fact that a semi-correlated Wishart matrix still belongs to a PE. Therefore, we just plug its joint unordered eigenvalue expression into (6), and compute the corresponding average. The result, along with numerical results confirming the tightness of our approximation, is reported in the following claim.

**Claim** **1.** *Assuming the kernel matrix to be complex central correlated Wishart, with* N≥K *degrees of freedom, with correlation matrix* Σ *of size*  *K*, *with distinct, ordered eigenvalues denoted by* $\sigma_{1}>\ldots >\sigma_{K}>0$*’s, i.e., ([10] Equation (94)),*

(12) $$\begin{array}{c} p\left(A\right)=\frac{{\left|A\right|}^{N-K}e^{-\mathrm{tr}(\Sigma^{-1}A)}}{{\left|\Sigma \right|}^{N}\prod_{\ell =1}^{K}{\left(\sqrt{\pi }\right)}^{K-1}\left(N-\ell \right)!}, \end{array}$$

*a suitable approximation for the MGF of* *W* *can be provided by* (2)*, with* 

$${\left[\Upsilon \left(s\right)\right]}_{i,j}=\left\{\begin{array}{c} \frac{(N-K)!}{{\left(\frac{1}{\sigma_{i}}+s\right)}^{N-K+1}},\;i=1,\ldots ,K,j=1,\\ \left(N-j\right)!\sigma_{i}^{N-j+1},\;i=1,\ldots ,K,j=2,\ldots ,K, \end{array}\right.$$

*while*

(13) $$C=\frac{{\left(-1\right)}^{K-1}\left(K-1\right)!}{\prod_{\ell =1}^{K}\sigma_{\ell }^{N-K+1}\left(N-\ell \right)!\prod_{i<j}\left(\sigma_{i}-\sigma_{j}\right)}.$$

The corresponding approximated PDF can be expressed as

(14) fW(t)=C∑i=1KκitN−1exp−tσiF11K−1;N;tσi,

for t>0 and with the normalizing constant in (13).

**Figure 2:**
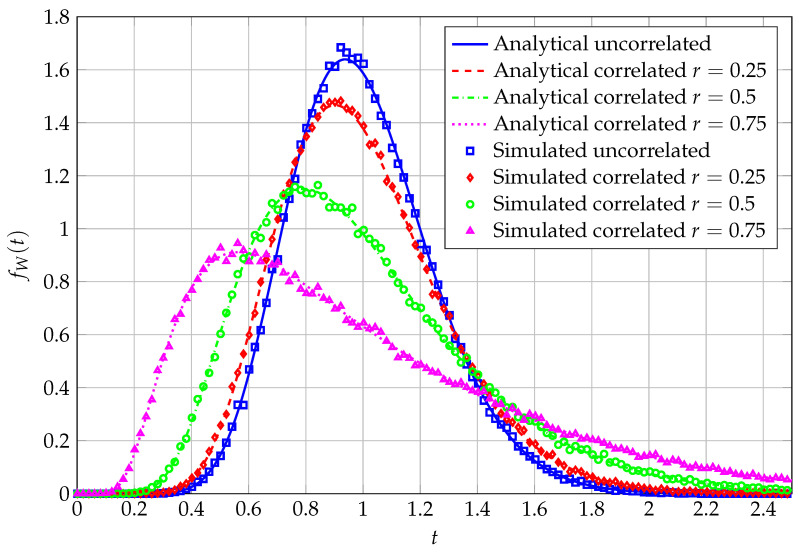
PDF of QF, analytical vs. simulated, for varying *r*, N=16, and K=4.

To enhance the tightness of our approximation, we plot in Figure 2 the simulated PDF, corresponding to a QF with a correlated Wishart kernel matrix, with an exponential correlation matrix, i.e., for r∈[0,1], ${\left[\Sigma \right]}_{i,j}=r^{\left|i-j\right|}$. Such a model, of relevance in applications within and beyond signal processing for wireless communications (see, e.g., [9] again), features all distinct eigenvalues; therefore, it matches the framework of our claim. For the fixed matrix size 4×16, PDF curves corresponding to various values of *r* are reported, ranging from negligible to very strong correlations. The baseline, uncorrelated case is reported as well. It is evident from the figure that, with an increasing value of the correlation coefficient, the PDF becomes less peaked and more heavy-tailed, whereas for a negligible to absent correlation, the curve peaks around higher values and shows an appreciably fast decay. In a wireless communications scenario, this corresponds to a higher (in the low-correlated case) versus lower (in the strongly correlated case) capability of exploiting the diversity order provided by system parameters incarnating the size of the kernel matrix *K* and, respectively, its degrees of freedom *N*.

**Figure 3:**
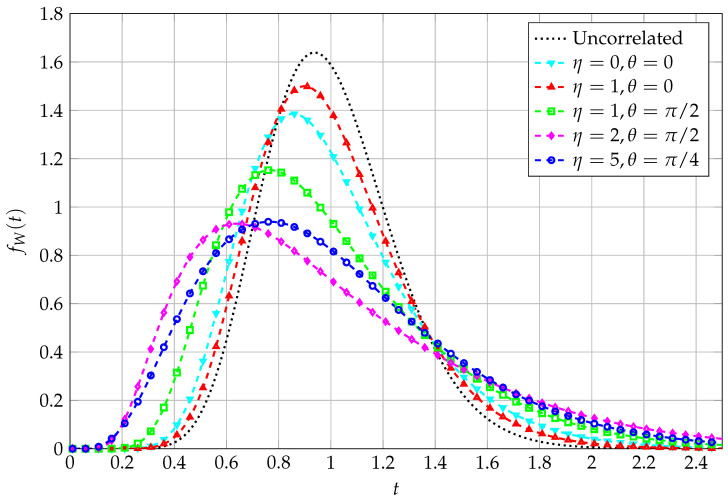
PDF of QF with angle-dependent correlation; N=16, K=4.

Simulations are then reported, along with the analytical approximation for corresponding system parameters, with reference to a different correlation model, where the entries of Σ depend on the geometry of a radio link with multiple transmit and multiple receive antenna elements [24]. We can therefore state that the correlation model is angle-dependent, via two parameters, say *θ* and η, whose ranges of variation are detailed in [24]. For such a scenario, the statistics of the QF, in terms of its PDF, are reported in Figure 3, where the matching between analytical and simulated curves is, once again, extremely satisfactory.

A further case of a unitarily invariant matrix of relevance to wireless applications is the product of independent complex Gaussian matrices with zero-mean iid entries, corresponding to multiple scattering phenomena on radio channels [11]. The MGF for this case is characterized below.

**Corollary** **2.** *Assuming the kernel matrix of the QF to correspond to the Gram matrix of the product of L independent factors, each given by, say, $H_{\ell }$, a $N_{\ell }\times N_{\ell -1}$ ($N_{0}=K$ for notational uniformity) complex zero-mean Gaussian matrix, with iid entries, the MGF of the QF can be written as per* (2)*, with*

[Υ(s)]i,j=G1,LL,11νL+1,…,ν1+i|1s,i=1,…,K,j=1,(ν1+K+i−j−1)!∏t=2L(νt+K−j)!,i=1,…,K,j=2,…,K,

*and*

$$C=\frac{{\left(-1\right)}^{\left(K-1)(K/2-1\right)}\left(K-1\right)!}{{\left(M\right)}^{K-1}\prod_{\ell =1}^{K}\prod_{t=0}^{L}\left(\nu_{t}+\ell -1\right)!}.$$

*Above, we have defined the set of excess degrees of freedom parameters, by the integers*

$$\nu_{\ell }=N_{\ell }-N_{0},$$

*with ℓ=1,…,L, while Gp.qm,na1,…,apb1,…,bq|z denotes the Meijer-G function ([21] 9.3).*

**Proof.**  The eigenvalue distribution of a product of independent matrices, distributed according our assumptions, is jointly distributed as [11]

(15) pΛ=G0,LL,0−νL,…,ν1+i−1|λj∏i<j(λi−λj)∏ℓ=1K∏t=0L(νt+ℓ−1)!.

Plugging (15) into (4) leads therefore to the sought expression for the MGF, with

[Υ(s)]i,j=∫0+∞e−sλG0,LL,0−νL,…,ν1+i−1|λdλ,i=1,…,K,j=1,∫0+∞λK−jG0,LL,0−νL,…,ν1+i−1|λdλ,i=1,…,K,j=2,…,K,

whose expression reverts to that reported in this corollary’s statement, by virtue of ([11] (A4), (A6), (A10)). □

**Figure 4:**
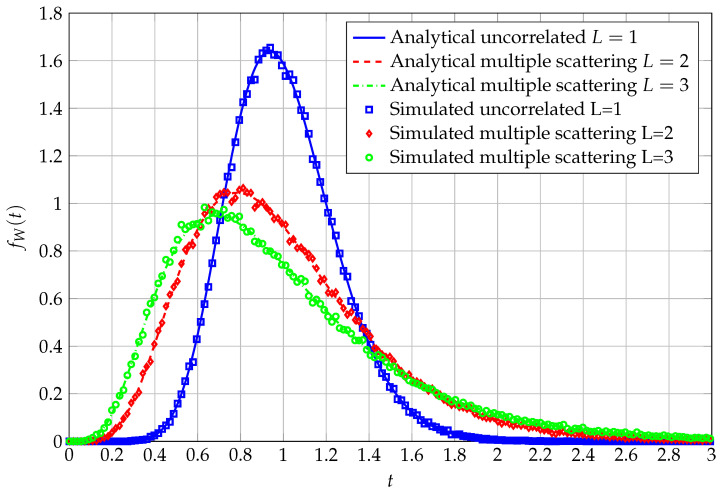
PDF of QF in the matrix product case, with varying values of *L*. N=16, K=4, S=8, Q=12.

**Figure 5:**
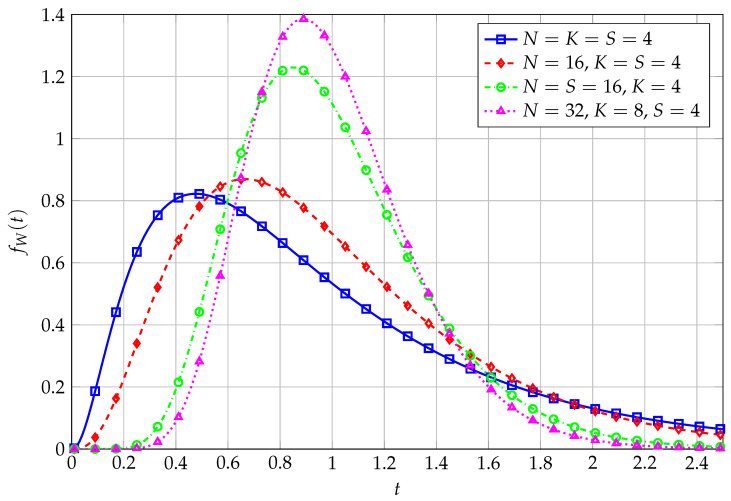
PDF of QF for L=2, for varying values of $\nu_{1}$ and $\nu_{2}$.

**Figure 6:**
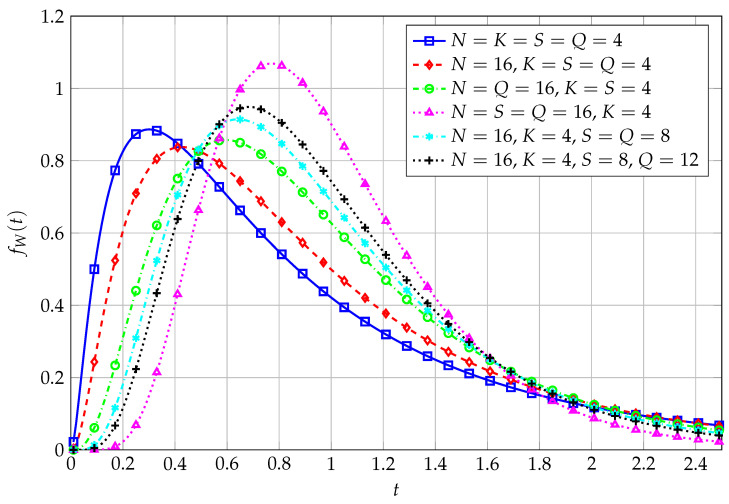
PDF of QF for L=3, for varying values of $\nu_{1}$, $\nu_{2}$, and $\nu_{3}$.

Recovering the PDF from the MGF is, in this last case, a more complex task than for previous cases. It is otherwise possible to resort to numerical Laplace inversion (via the Talbot method [25]), leading to the data being displayed as in Figure 4, Figure 5 and Figure 6.

In Figure 4, the PDF of the QF, obtained via Laplace inversion with Talbot method, for three different values of *L* is reported, along with corresponding simulated data. In particular, for L=1, $N_{0}=4$ and $\nu_{1}=12$ are exploited to generate the curves. In the case of L=2, instead, we use $N_{0}=4$, $\nu_{1}=4$ and $\nu_{2}=12$. Finally, given L=3, $N_{0}=4$, $\nu_{1}=4$, $\nu_{2}=8$ and $\nu_{3}=12$ are adopted. The higher the value of *L*, the higher the generated dependence among the entries of the overall matrix product, and the lower, as a consequence, the peak of the corresponding PDF.

A few remarks are in order: for L=1, the model reverts to that of an uncorrelated Wishart matrix, as per (7). Otherwise, for low values of *L*, a generalized hypergeometric function can be exploited, rather than the Meijer one, according to

(16) G1,LL,11νL+1,…,ν1+i|1s=(ν1+i−1)!∏ℓ=2Lνℓ!F0L(νL+1,…,ν1+i|−s),

whose convergence is discussed in detail, for example, in [26]. Equation (16) becomes, for L=1, $\frac{(\nu_{1}+i-1)!}{{\left(1+s\right)}^{\nu_{1}+i}}$, while, for L=2,

(ν1+i−1)!ν2!F02(ν2+1,ν1+i|−s),

with F02 as a generalized hypergeometric function ([22] Ch.13.1).

The newly obtained MGF is inverted with Talbot method for the case of three matrix factors (L=3) as well, and the corresponding results are contrasted with simulated data, and reported, for varying values of the set of excess degrees of freedom, in Figure 6.

Among the results of these three discussed particular cases, the one in Corollary 1 can be exploited in advanced wireless communication settings for 6G and beyond ([27,28,29,30] and references therein). The illustration thereof is the subject of the next section.

## 3. Application to Spectrum Sensing

A possible application framework of our newly derived results, for the uncorrelated Wishart case, is within CRN [31,32,33]. Specifically, the statistics of a QF can help in detecting the presence of a primary source of signal, upon the absence of which the channel is considered clear to for secondary user access. Let us focus on a setting, where a receiver equipped with *K* antennas collects *N* time samples from each antenna. We denote by $y\left(n\right)={\left[y_{1}\left(n\right),\ldots ,y_{K}\left(n\right)\right]}^{T}$ the K×1 received vector at time $n\in \left\{1,\ldots ,N\right\}$, with generic entry $y_{k}\left(n\right)$ denoting the discrete baseband complex sample at the *k*-th receive antenna. We tackle the sensing problem as a binary hypothesis test. Under $H_{0}$, namely in the absence of a primary signal, $y_{k}\left(n\right)$ is a vector of complex Gaussian noise samples with zero mean and variance $\sigma_{v}^{2}$

(17) $${y\left(n\right)|}_{H_{0}}=v\left(n\right)$$

where $v\left(n\right)∼N_{C}\left(0_{K\times 1},\sigma_{v}^{2}I_{K\times K}\right)$. Under $H_{1}$, instead, both primary signal and noise are present; therefore

(18) $${y\left(n\right)|}_{H_{1}}=x\left(n\right)+v\left(n\right)=hs\left(n\right)+v\left(n\right)$$

where s(n) is the transmitted signal sample, modeled without loss of generality as Gaussian distributed with zero mean and variance $\sigma_{s}^{2}$, while h is the K×1 unknown complex channel vector. The channel is assumed to be memoryless, and constant during the detection time. Under $H_{1}$, we define the Signal-to-Noise Ratio (SNR) at the receiver as

(19) $$\rho ≜\frac{{E\;∥x\left(n\right)∥}^{2}}{{E\;∥v\left(n\right)∥}^{2}}=\frac{\sigma_{s}^{2}}{\sigma_{v}^{2}}\frac{{∥h∥}^{2}}{K}.$$

The detector collects the received samples in the K×N matrix

(20) $$Y≜\left[y(1)\cdots y(N)\right]=\mathrm{hs}+V$$

where $s≜\left[s(1)\cdots s(N)\right]$ is a 1×N signal vector and $V≜\left[v(1)\cdots v(N)\right]$ is a K×N noise matrix. The sample covariance matrix R of the received samples is therefore given by

(21) $$R≜\frac{1}{N}YY^{H}.$$

Hereafter, we shall reference the spectral decomposition of $R=U\Lambda U^{H}$, with U denoting the random unitary matrix of the eigenvectors, and Λ the diagonal matrix of the random eigenvalues, which are without loss of generality, denoted in non-increasing order as $\lambda_{1}\geq \cdots \geq \lambda_{K}\geq 0$. It is apparent from our assumptions that the matrix R is, in the null hypothesis, complex central uncorrelated Wishart-distributed, according to (7).

The test statistic, employed by the detector to discriminate between the null hypothesis $H_{0}$ and the presence of primary signal $H_{1}$, is denoted by *T*; in order to take a decision, the detector compares it against a pre-defined threshold *t*: if T>t, it selects $H_{1}$; otherwise, it selects $H_{0}$. As a consequence, the *probability of false alarm*$P_{fa}$ is defined as

(22) $$P_{fa}=\mathrm{Pr}\left(T>t|H_{0}\right)$$

and the *probability of detection* $P_{d}$ as

(23) $$P_{d}=\mathrm{Pr}\left(T>t|H_{1}\right).$$

The sensing scheme we focus on was introduced in [33]; such a method makes use of the sample eigenvector corresponding to the largest eigenvalue (i.e., $\lambda_{1}$) of (21) under $H_{1}$, corresponding to the received sample matrix in (20), and it is therefore referred to as EigenVEctor-aided test (EVE), with the statistic given by

(24) $$T_{\mathrm{EVE}}≜\frac{\lambda_{1}+Mu_{1}^{H}Ru_{1}}{\sigma_{v}^{2}\left(M+1\right)},$$

where *M* is the number of auxiliary slots exploited to estimate R under $H_{1}$, namely to obtain the sample eigenvector estimate $u_{1}$, corresponding to $\lambda_{1}$.

In absence of auxiliary data, $T_{\mathrm{EVE}}$ reduces to the widely adopted Roy’s Largest Root Test (RLRT) [34], with the statistic

(25) $$T_{\mathrm{RLRT}}≜\frac{\lambda_{1}}{\sigma_{v}^{2}};$$

for this reason, EVE can be seen as a heuristic improvement of the RLRT.

On the other hand, as *M* grows, the effect of the secondary data prevails upon the presence of the largest root $\lambda_{1}$ in the enumerator of (24). With this in mind, we postulate the availability of a sufficient number of secondary data to approximate $T_{\mathrm{EVE}}$ with a test where only the QF at the enumerator is considered for SS purposes; the resulting test, based on the statistics of

$$u_{1}^{H}Ru_{1},$$

will be hereafter referred to asthe Quadratic Form Test (QFT), with the statistic given by

(26) $$T_{\mathrm{QFT}}≜\frac{u_{1}^{H}Ru_{1}}{\sigma_{v}^{2}}.$$

It is immediately noticeable that the computation of the $P_{fa}$ (22) for the test above can be carried out by direct application of our Corollary 1. Indeed, (26) is nothing but a properly scaled (by the noise variance) QF, and, upon integration of (11) against a pre-defined threshold, the sought performance index is obtained.

Our interest in QF to approximate EVE arises from the possibility of getting a closed-form statistical characterization of SS performance, in an easier way than for EVE itself. On top of that, we recall that, when secondary data are made available, a performance gap in favor of $T_{\mathrm{EVE}}$ over many classically adopted SS tests has been numerically observed (see [33,35]).

**Figure 7:**
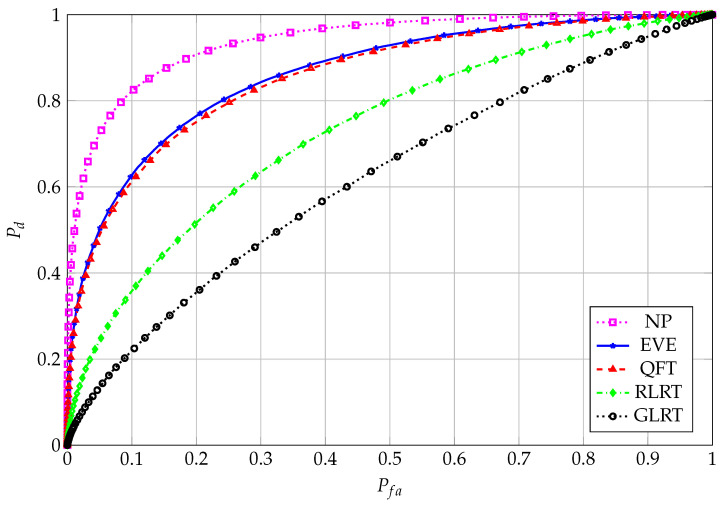
ROC curve; N=64, K=8, M=4 for EVE and QFT; SNR=−5 dB.

**Figure 8:**
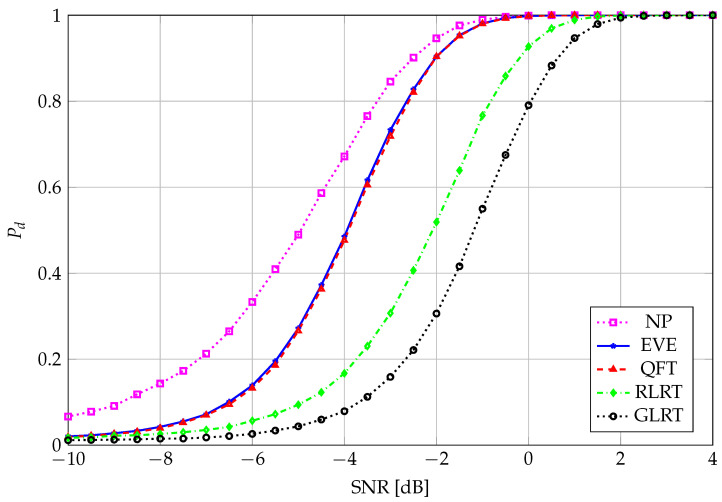
Performance curve; $P_{d}$ vs. SNR; N=64, K=8, M=4 for EVE and QFT; $P_{fa}=0.01$.

We enhance the performance gain of EVE (and, correspondingly, of QFT) in Figure 7 and Figure 8, where the corresponding Receiver Operating Characteristic (ROC) curve and the performance curve, i.e., $P_{d}$ vs. SNR, are shown, respectively, for the goodness of such an approximation. The ROC and performance curve are shown also for the Generalized Likelihood-Ratio Test (GLRT) and the Neyman–Pearson (NP) reference test. For the sake of clarity, we recall that the GLRT test statistic is given by [36]

(27) $$T_{\mathrm{GLRT}}=\frac{\lambda_{1}}{\frac{1}{K}\mathrm{tr}\left(R\right)},$$

while the Neyman–Pearson test, which requires the exact knowledge of both channel vector h and noise variance $\sigma_{v}^{2}$, under the assumption of independent Gaussian samples, is given by [36]

(28) $$T_{\mathrm{NP}}=\frac{{\left(\pi \sigma_{v}^{2}\right)}^{NK}\exp\left(-N\mathrm{tr}(R\Gamma^{-1})\right)}{{\left(\pi^{K}\left|\Gamma \right|\right)}^{N}\exp\left(-\frac{N\mathrm{tr}(R)}{\sigma_{v}^{2}}\right)}$$

where

(29) $$\Gamma =\sigma_{v}^{2}I_{K\times K}+\sigma_{s}^{2}hh^{H}.$$

## 4. Conclusions

We have provided new closed-form statistics for QF in random kernel matrices from polynomial ensembles. Albeit rigorously valid for unitarily invariant matrices, our result led also to a tight approximation for the MGF of a QF with a non-unitarily invariant matrix kernel. An application of our result in CRN is also reported. Indeed, it is shown that the performance of an effective SS scheme can be suitably approximated by a statistical test formulated in terms of a QF with an uncorrelated Wishart kernel. Further investigations on matrices from polynomial ensembles, beyond unitarily invariance, are subject to ongoing work.

## Data Availability

The data that support the findings of this study are available from the first author, D.G.R., upon reasonable request.

## Abbreviations

The following abbreviations are used in this manuscript:

EVE	EigenVEctor-aided test
GLRT	Generalized Likelihood-Ratio Test
MDPI	Multidisciplinary Digital Publishing Institute
MGF	Moment Generating Function
NP	Neyman–Pearson
PDF	Probability Density Function
PE	Polynomial Ensemble
QF	Quadratic Form
QFT	Quadratic Form Test
RLRT	Roy’s Largest Root Test
ROC	Receiver Operating Characteristic
SNR	Signal-to-Noise Ratio
SS	Spectrum Sensing
